# Acute Abdominal Pain after Intercourse: Adrenal Hemorrhage as the First Sign of Metastatic Lung Cancer

**DOI:** 10.1155/2014/612036

**Published:** 2014-07-13

**Authors:** Jeremy Wang, Clifford D. Packer

**Affiliations:** ^1^Case Western Reserve University School of Medicine, 10900 Euclid Avenue, Cleveland, OH 44106, USA; ^2^Louis Stokes Cleveland VA Medical Center, 10701 East Boulevard, Cleveland, OH 44106, USA

## Abstract

Although the adrenal glands are a common site of cancer metastases, they are often asymptomatic and discovered incidentally on CT scan or autopsy. Spontaneous adrenal hemorrhage associated with metastatic lung cancer is an exceedingly rare phenomenon, and diagnosis can be difficult due to its nonspecific symptoms and ability to mimic other intra-abdominal pathologies. We report a case of a 65-year-old man with a history of right upper lobectomy seven months earlier for stage IB non-small cell lung cancer who presented with acute abdominal pain after intercourse. CT scan revealed a new right adrenal mass with surrounding hemorrhage, and subsequent FDG-PET scan confirmed new metabolic adrenal metastases. The patient's presentation of abdominal pain and adrenal hemorrhage immediately after sexual intercourse suggests that exertion, straining, or increased intra-abdominal pressure might be risk factors for precipitation of hemorrhage in patients with adrenal metastases. Management includes pain control and supportive treatment in mild cases, with arterial embolization or adrenalectomy being reserved for cases of severe hemorrhage.

## 1. Introduction

The adrenal glands are a common site of cancer metastases, with 27% of cancer patients found to have adrenal metastases on autopsy in one study [1]. Primary sites include carcinoma of the breast, lung, and kidney [2]. Up to 40% of patients with non-small cell lung cancer develop adrenal metastases as the carcinoma progresses [3]. However, adrenal metastases are typically asymptomatic and are often discovered incidentally on abdominal CT [4]. Spontaneous adrenal hemorrhage with any underlying malignant mass is a rare phenomenon [5–7], and adrenal hemorrhage in cases of metastatic lung cancer is exceedingly rare; a literature review by Marti et al. of 133 cases of hemorrhagic adrenal masses revealed pheochromocytoma and pseudocysts as the most common pathologic diagnoses, with only 9 (6.8%) cases of metastatic lung cancer [6]. To our knowledge, our case of a 65-year-old man with adrenal hemorrhage from metastatic lung cancer is only the 27th reported worldwide [8]. Although there are a few case reports of adrenal hemorrhage associated with straining and heavy lifting [6], ours is the first to describe malignant adrenal hemorrhage in the setting of sexual intercourse.

## 2. Case Presentation

A 65-year-old Caucasian man, a Vietnam veteran and retired truck driver, presented to the emergency room three hours after onset of severe right lower quadrant and flank pain that had started a few minutes after sexual intercourse that evening. The pain was sharp in quality, constant, and nonradiating, and there were no aggravating or alleviating factors. The patient developed nausea and vomited once shortly after the onset of pain. There were no fevers, chills, dysuria, hematuria, hematemesis, melena, or hematochezia. Past medical history was significant for Crohn's disease with two remote ileocolic resections and a right upper lobectomy seven months earlier for stage IB non-small cell lung cancer. On examination, the patient was afebrile with a pulse of 81 beats per minute, respiratory rate of 16 per minute, and blood pressure of 160/75 mmHg. Height was 68 inches, weight 203 pounds, BMI 31.0. Physical examination was unremarkable except for marked tenderness in the right upper and lower abdomen, with voluntary guarding but no rigidity or rebound tenderness.

**Figure 1:**
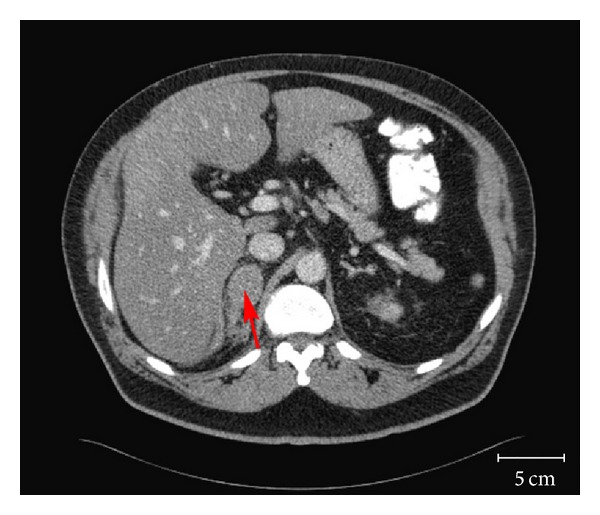
Abdominal computed tomography (CT) scan revealing a 3.6 × 2.7 cm mass (red arrow) involving the right adrenal gland.

**Figure 2:**
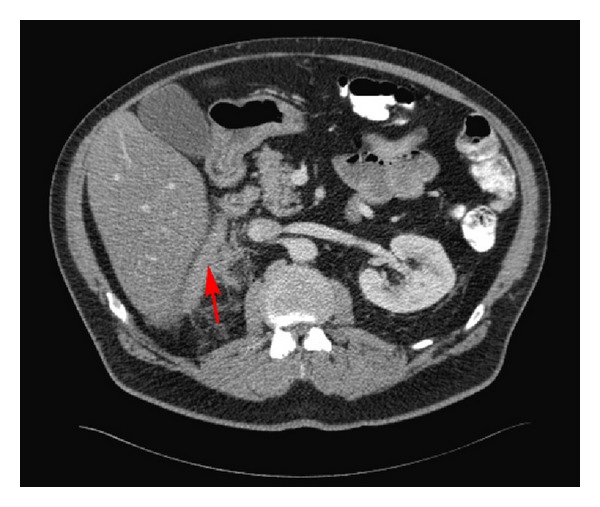
Abdominal computed tomography (CT) scan revealing streaky densities in the fat (red arrow) adjacent to the right adrenal gland; findings are consistent with hemorrhage.

**Figure 3:**
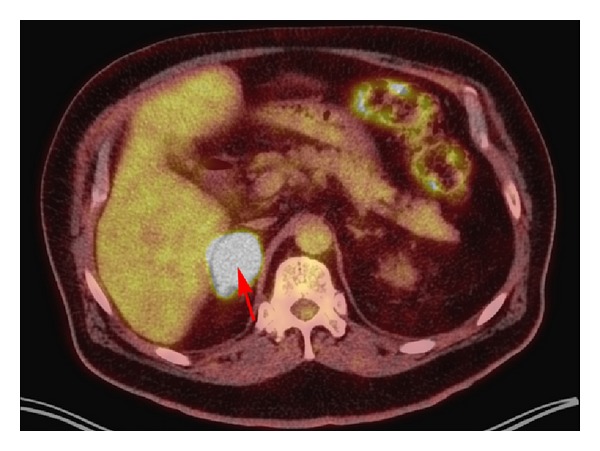
18F-fluorodeoxyglucose positron emission tomography (FDG-PET) scan revealing an intensely metabolic (SUV_max⁡_ = 19.8) right adrenal mass (red arrow), confirming new adrenal metastasis.

Initial laboratory data revealed a hemoglobin of 12.4 g/dL and hematocrit of 37.6%, white blood cell count of 9.03 × 10^9^/L, and platelet count of 259 × 10^9^/L. Plasma cortisol was normal at 10.5 *μ*g/dL. An abdominal computed tomography (CT) scan revealed a 3.6 × 2.7 cm right adrenal mass (Figure 1), which was not present on an abdominal CT scan done four months earlier. A band of mild increased density seen in the fat situated between the posteromedial aspect of the right lobe of the liver and the right adrenal gland and streaky densities seen in the fat adjacent to the posterior and inferior aspects of the right adrenal gland were also new and consistent with hemorrhage (Figure 2). At this point, it was assumed that the adrenal mass with hemorrhage was most likely the cause of the acute abdominal pain, but the etiology of the mass was still unclear. The patient's abdominal pain was controlled with morphine and hydromorphone. His vital signs and hemoglobin remained stable overnight, and he did not require blood transfusion. The next day, he complained of vision loss and his examination suggested visual field defects. Magnetic resonance imaging (MRI) of the head revealed probable brain metastases. 18F-fluorodeoxyglucose positron emission tomography (FDG-PET) scan on hospital day number 2 revealed increased 18F-FDG uptake in the right adrenal gland with a maximal standardized-uptake-value (SUVmax) of 19.8, confirming new metabolic adrenal metastasis (Figure 3). PET MRI performed 3 days after admission showed evidence of both regional and distant metastases, with bilateral hilar and mediastinal lymphadenopathy, left lower lobe and left supraclavicular nodules, right temporooccipital and right frontoparietal brain metastases, and a hemorrhagic right adrenal metastasis. All of these findings were new compared with a chest CT from 3 months before, which showed only postsurgical right upper lobe changes, mild right paratracheal and aortopulmonary lymphadenopathy, two stable subcentimeter left lower lobe nodules, and unremarkable adrenal glands. These new findings strongly suggested metastatic lung cancer as the cause of both the brain lesions and the right adrenal mass with hemorrhage. Six days after admission, the patient underwent craniotomy and resection of brain metastases. He was subsequently treated with whole-brain radiation therapy, followed by palliative chemotherapy starting 6 weeks later.

## 3. Discussion

Presenting symptoms of adrenal hemorrhage are typically nonspecific and include sudden abdominal, chest, flank, or back pain, nausea and vomiting, hypotension/shock, tachycardia, and fever [4]. Thus, adrenal hemorrhage is a challenging diagnosis because symptoms can mimic many other abdominal pathologies. Fortunately, our patient experienced a relatively mild hemorrhagic episode. He presented with acute abdominal pain, nausea, and vomiting but did not develop hypotension and did not require fluid resuscitation or blood transfusions. Of note, however, several case reports have described patients who presented with critical anemia, hypotension, or even sudden death [4–6]. Thus, given its potentially dangerous consequences, adrenal metastasis with hemorrhage needs to be considered in the differential diagnosis of any patient presenting with sudden onset abdominal pain accompanied by anemia or hypotension, especially if they have a history of cancer. Computed tomography scanning is highly sensitive and specific in the diagnosis of adrenal hemorrhage and therefore remains the preferred imaging modality. CT evidence of adrenal hemorrhage is variable: it may present as a nonhomogenous mixed density adrenal mass with extensive perirenal changes, a rapidly enlarging adrenal mass, or extensive retroperitoneal hemorrhage [9, 10].

Our patient's unexpected presentation of acute abdominal pain and adrenal hemorrhage immediately after sexual intercourse suggests the possibility that exertion, straining, or increased intra-abdominal pressure might be risk factors for precipitation of hemorrhage in patients with adrenal metastases. Marti et al. [6] describe a series of six patients who presented with spontaneous adrenal hemorrhage that appeared to be associated with a neoplasm. Of the six, five presented with acute flank pain, and two, a 47-year-old man with adrenocortical carcinoma and a 62-year-old man with a noncancerous adrenal mass, presented with pain after heavy lifting. To our knowledge, ours is the first case of spontaneous adrenal hemorrhage after sexual intercourse to be described in the literature. In view of these reports, a history of activities immediately preceding the onset of abdominal pain should be taken and carefully considered.

Due to its rarity, a consensus on the optimal approach and management of adrenal hemorrhage has not yet been reached. Our patient's case was relatively mild; general surgery was consulted out of concern for continuing hemorrhage that might require surgical intervention, but ultimately only supportive treatment and pain control were required. In other cases, management varied according to the severity of the hemorrhage. Most patients were managed with supportive treatment, including volume resuscitation, blood transfusions, and observation [3, 6, 7]. There is one report of a patient treated with transcatheter embolization of the middle adrenal artery [11]. In another case of severe hemorrhage, adrenalectomy was required [12].

Physicians should consider adrenal hemorrhage in the differential diagnosis of any cancer patient presenting with acute abdominal pain, especially if accompanied by significant anemia, hypotension suggesting possible massive hemorrhage, or signs of adrenal insufficiency. Activities that increase intraabdominal pressure, such as heavy lifting and sexual intercourse, may be risk factors for spontaneous adrenal hemorrhage. Early recognition and prompt CT imaging can reduce morbidity and in some cases may lead to life-saving interventions for these patients.
